# Comparative effects of recombinant human brain natriuretic peptide and dobutamine on acute decompensated heart failure patients with differsent blood BNP levels

**DOI:** 10.1186/1471-2261-14-31

**Published:** 2014-03-04

**Authors:** Hai-Yan Pan, Jian-Hua Zhu, Yong Gu, Xiao-Hong Yu, Min Pan, Hong-Yin Niu

**Affiliations:** 1 Department of Cardiology, Affiliated Hospital of Nantong University, and Institute of Cardiovascular Research, Nantong University, Jiangsu 226001, China; 2 Department of Echocardiography, Affiliated Hospital of Nantong University, Jiangsu, China

**Keywords:** Acute decompensated heart failure, Recombinant human brain natriuretic peptide, Dobutamine

## Abstract

**Background:**

Recombinant human B-type natriuretic peptide (rhBNP) has been indicated for the treatment of acute decompensated heart failure (ADHF). However, the therapeutic efficacy of intravenous rhBNP is not always satisfactory in patients with extremely high blood BNP levels. In this study, we evaluated the effects of rhBNP on patients with different BNP levels.

**Methods:**

One hundred and five patients with ADHF whose left ventricular ejection fraction (LVEF) was <40%, were assigned to a high BNP group (BNP ≤ 3000 pg/mL) or an extra-high BNP group (BNP > 3000 pg/mL) , depending on their admission plasma BNP levels. Each group was then subdivided into rhBNP or dobutamine subgroups according to intravenous administration with either rhBNP or dobutamine for 24-72h. In the high BNP group, 58 patients were randomized to subgroup rhBNP (n = 28) and subgroup dobutamine (n = 30). In the extra-high BNP group, 47 patients were randomized to subgroup rhBNP (n = 24) and subgroup dobutamine (n = 23). The effects of rhBNP and dobutamine on patients in the high and extra-high BNP groups were compared.

**Results:**

In the high BNP group, rhBNP was more efficient than dobutamine at improving NYHA classification (*P* < 0.05), decreasing plasma BNP levels (*P* < 0.05), increasing LVEF (*P* < 0.05), and reducing hospital length of stay (*P* < 0.05). However, rhBNP displayed no superior therapeutic efficacy to dobutamine in the extra-high BNP group. Adverse cardiovascular events in patients treated with rhBNP were similar to adverse events in patients treated with dobutamine in both the high and extra-high BNP groups.

**Conclusions:**

rhBNP was more efficient than dobutamine at improving heart function in patients with ADHF when plasma BNP was ≤3000 pg/mL. However, rhBNP treatment showed no advantages over dobutamine when plasma BNP reached extremely high levels (>3000 pg/mL).

**Trial registration:**

ClinicalTrials.gov Identifier: NCT01837849.

## Background

Acute decompensated heart failure (ADHF) is associated with significant morbidity and mortality and remains a common cause of hospitalization worldwide. As a result, therapies targeting both symptoms and cardiovascular outcomes are greatly needed. Two such treatments are dobutamine and recombinant human brain natriuretic peptide (rhBNP).

Dobutamine is a synthetic catecholamine that acts mainly through stimulation of β1-receptors and partly through β2-receptors to produce dose-dependent positive inotropic and chronotropic effects [1]. It has long been used as a potent positive inotrope for the management of severe heart failure. rhBNP is a recombinant formulation of BNP that is identical to the endogenous hormone released from the cardiac ventricle in response to increases in wall stress, hypertrophy, and volume overload [2]. It is a potent vasodilator that is proven to rapidly reduce cardiac filling pressures and improve dyspnea in patients with ADHF [3]. In 2005, rhBNP was recommended in the ADHF treatment guidelines by the European Society of Cardiology [4].

Some evidence suggests that rhBNP is more effective than dobutamine in the treatment of heart failure [5-7]. However, in our clinical work, we have found that the effects of rhBNP are not always satisfactory in patients with extremely high blood BNP levels, with the heart function of some patients not improved until rhBNP is replaced by dobutamine. Although comparison of rhBNP and dobutamine in ADHF patients has been reported in several studies [5-7], no comparative study has been performed on ADHF patients with different plasma BNP levels. Thus, the purpose of this study was to determine the therapeutic efficacy of rhBNP in ADHF patients with different plasma BNP levels in comparison to that of dobutamine in order to provide valuable information for the treatment of heart failure.

## Methods

### Patient selection

Between January 2011 and June 2012, patients with ADHF who required hospitalization were enrolled in the study. The mean age was 66 ± 13 years (range = 29–88). The ADHF etiology included idiopathic dilated cardiomyopathy (IDC), coronary heart disease (CHD), fulminant viral myocarditis (FVM), hyperthyroid heart disease (HHD), and valvulopathy. All patients were classified as New York Heart Association (NYHA) class III–IV and had an ejection fraction of <40%. Exclusion criteria included intravenous administration with rhBNP or dobutamine in the 2 weeks before study entry, acute myocardial infarction, significant valvular stenosis, serious ventricular arrhythmia (frequent ventricular premature beat of >5 bpm, nonsustained and sustained ventricular tachycardia), blood pressure <95/60 mmHg or >140/90 mmHg, shock, hypovolemia, and hepatic or renal impairment [alanine aminotransferase or aspartate aminotransferase >2 times the upper limit of the normal value, serum creatinine >177 μmol/L (2.0 mg/dl) for man or >146 μmol/L (1.7 mg/dl) for women]. In addition, pregnant and lactating women, those who had to change medication due to adverse events during the study were also excluded. All patients provided informed consent, and the Ethics Committee of Affiliated Hospital of Nantong University approved the protocol.

### Study design

Previous studies evaluating the correlation between BNP level and heart failure severity used 3000 pg/mL as the cut-off for extremely high BNP elevation [8,9]. In this open-label prospective cohort study we also used value of 3000 pg/mL (30 times the upper normal range) as the cut-off for stratifying patients into high and extra BNP groups. Enrolled patients were stratified into a high BNP group (BNP ≤ 3000 pg/mL) or extra-high BNP group (BNP > 3000 pg/mL) depending on their admission plasma BNP levels. Each group was then subdivided into two subgroups (rhBNP or dobutamine) according to the intravenous administration of either rhBNP or dobutamine. Patients in the high and extra-high BNP groups were randomly assigned to rhBNP or dobutamine subgroups using a computer-generated random number table. In total, 58 patients were enrolled in the high BNP group, of which 28 were randomized to the rhBNP subgroup and 30 were randomized to the dobutamine subgroup. In addition, 47 patients were enrolled in the extra-high BNP group, of which 24 were randomized to the rhBNP subgroup and 23 were randomized to the dobutamine subgroup.

### Procedure

In addition to conventional heart failure therapy, patients in the rhBNP and dobutamine subgroups received rhBNP (marketed as Xinhuosu, Chengdu Rhodiola Bio-Pharmacy, Chengdu, China) and dobutamine (Shanghai No. 1 Biochemical & Pharmaceutical, Shanghai, China) infusion, respectively, for a period of 24–72 h. rhBNP was administered as an intravenous bolus of 1.5 μg/kg followed by continuous infusion in doses of 0.0075–0.01 μg/kg/min according to each patient’s clinical status. Patients in the dobutamine subgroup initially received 2.5 μg/kg/min dobutamine and were up-titrated to 10 μg/kg/min if hemodynamic goals were not achieved.

Plasma creatinine and BNP levels were measured at baseline and 5 days after the start of the study. Creatinine was detected by a creatinine assay kit (Abcam, MA, USA). BNP levels were determined with the ARCHITECT BNP Reagent Kit (Abbott Laboratories, IL, USA). Left ventricle end diastolic dimension (LVEDD) and left ventricular ejection fraction (LVEF) were measured by one observer blinded to the clinical data with a GE Vivid E9 ultrasound scanner (GE Healthcare, Horten, Norway) at the time of blood sample drawing.

### Assessment of efficacy

The drug therapeutic efficacy was assessed as improvement of functional class; changes in BNP levels, LVEDD, and LVEF from baseline to fifth day after the beginning of treatment. According to the assessed dyspnea and systemic symptoms, the functional improvement was graded as "excellent" (NYHA class was improved ≥2 functional classes), "improved" (NYHA class was improved 1 functional class) or "failed" (NYHA class remained the same or deteriorated). The overall effective rate was calculated as (patients with "excellent" + patients with "improved"/all patients) × 100%.

In addition, the length of hospitalization and the effects of the study drugs on blood pressure, heart rate (HR), blood creatinine, and cardiovascular adverse events during infusion were also analyzed.

### Statistical analysis

Statistical analysis was performed with SigmaStat 3.5 (Systat Software, Point Richmond, CA, USA) statistical software. Quantitative data are presented as mean ± standard deviation (SD), while categorical data are presented as numbers. Comparisons between the two groups were made using a two-sample *t*-test for continuous variables and chi-squared test or Fishe exact test for categorical data. Differences between baseline and treatment values were assessed using a paired *t*-test. *P* < 0.05 was considered significant.

## Results

### Baseline characteristics

In the extra-high BNP group, one patient who was assigned to the rhBNP subgroup had to withdraw from the study because of symptomatic hypotension 3 h after rhBNP administration; thus, 23 patients in the rhBNP subgroup finished this study. Basal blood pressure and plasma creatinine and baseline demographics and medical history information for patients in the high and extra-high BNP groups are summarized. Basal SBP level tended to be lower in the extra-high BNP group than in the high BNP group (P = 0.079), while plasma creatinine was significantly higher in the extra-high BNP group (P < 0.01) (Table 1). However, the baseline characteristics of the patients and heart failure medications in patients randomized to the rhBNP subgroup or the dobutamine subgroup were well balanced in both the high and extra-high BNP groups (Table 2).

**Table 1:** Baseline blood pressure and plasma creatinine in the high and Extra-high BNP groups

**Parameters**	**High-BNP group**	**Extra-high BNP group**	P value*
	**(n = 58)**	**(n = 46)**	
SBP (mmHg)	121.0 ± 12.3	116.7 ± 12.5	0.079
DBP (mmHg)	72.9 ± 7.2	71.3 ± 6.5	0.229
Cr (μmol/L)	81.6 ± 30.7	97.0 ± 27.1	0.009

*Unpaired *t* test.

*Abbreviations*: *BNP* brain natriuretic peptide, *Cr* creatinine, *DBP* diastolic blood pressure, *SBP* systolic blood pressure.

**Table 2:** Baseline characteristics and treatment information

**Characteristics**	**High BNP group**			**Extra-high BNP group**		
	**Dobutamine (n = 30)**	**rhBNP (n = 28)**	**P value**	**Dobutamine (n = 23)**	**rhBNP (n = 23)**	**P value**
Age (y)	63 ± 13	65 ± 13	0.495†	68 ± 14	67 ± 12	0.709†
Male/female	19/11	18/10	0.843‡	15/8	16/7	0.935‡
HF etiology						
IDC	13	11	0.963‡	10	12	0.768‡
CHD	8	10	0.645‡	10	8	0.763‡
FVM	2	1	1.000§	0	1	1.000§
HHD	1	2	0.605§	0	1	1.000§
Valvulopathy	6	4	0.732§	3	1	0.608§
NHYA			0.645‡			0.489§
III	8	10		0	2	
IV	22	18		23	21	
Concomitant medications						
ACEI	14	16	0.593‡	14	12	0.766‡
ARB	12	7	0.349‡	5	8	0.513‡
Digitalis	24	23	0.899‡	18	20	0.699§
β-blockers	11	14	0.448‡	4	6	0.722§
Spironolactone						
20 (mg/d)	15	18	0.405‡	11	6	0.324‡
40 (mg/d)	10	8	0.914‡	10	12	0.768‡
Average dosage	28.0 ± 10.0	26.2 ± 9.4	0.500†	29.5 ± 10.2	33.3 ± 9.7	0.243†
Furosemide (mg/d)						
20 (mg/d)	12	15	0.440‡	7	4	0.328§
40 (mg/d)	11	8	0.707‡	11	9	0.766‡
80 (mg/d)	2	3	0.665§	3	5	0.699§
Average dosage	33.6 ± 17.0	33.1 ± 19.5	0.919†	39.0 ± 19.5	46.7 ± 22.8	0.267†

†Unpaired *t*-test; ‡Chi-square test; §Fisher exact test.

*Abbreviations*: *ACEI* angiotensin-converting enzyme inhibitor, *ARB* angiotensin II receptor blocker, *BNP* brain natriuretic peptide, *CHD* coronary heart disease, *HF* heart failure, *HHD* hyperthyroid heart disease, *IDC* idiopathic dilated cardiomyopathy, *NHYA* New York Heart Association, *rhBNP* recombinant human brain natriuretic peptide. Values are expressed as number or mean ± SD.

### Changes in NYHA class

Functional improvements, as assessed by NYHA class, were observed so as to evaluate the therapeutic efficacy of the treatments. Both rhBNP and dobutamine could effectively improve patient cardiac function. In the high BNP group, rhBNP was significantly more efficacious than dobutamine in improving NYHA class. The proportion of patients showing a functional improvement of at least 1 grade in NYHA class was significantly greater in the rhBNP subgroup (92.86%, 26/28) than in the dobutamine subgroup (70%, 21/30) (*P* < 0.05). However, in the extra-high BNP group, the overall effective rates of the two subgroups did not differ significantly [56.52% (13/23) vs. 65.22 (15/23) (*P* > 0.05)] (Table 3).

**Table 3:** Changes in NYHA class from baseline to day 5 after treatment

**Groups**	**Dobutamine**					**rhBNP**				
	**Total**	**Ex**	**Im**	**Fa**	**OER (%)**	**Total**	**Ex**	**Im**	**Fa**	**OER (%)**
High BNP	30	12	9	9	21 (70.00)	28	9	17^*^	2	26 (92.86)^*^
Extra-high BNP	23	6	9	8	15 (65.22)	23	5	8	10	13 (56.52)

*Abbreviations*: *Ex* Excellent, *Fa* failed, *Im* improved, *OER* overall effective rate. Other abbreviations are same as in Table 2. ^*^P < 0.05 versus (Fisher exact test) dobutamine.

### Changes in blood BNP, LVEDD and LVEF in the high BNP group

Changes in blood BNP, LVEDD, and LVEF were analyzed to assess the therapeutic efficacy. In the high BNP group, both rhBNP and dobutamine were able to significantly reduce the plasma BNP levels (*P* < 0.01), decrease LVEDD (*P* < 0.01), and increase LVEF (*P* < 0.01). Compared to dobutamine, rhBNP was significantly more efficacious in reducing plasma BNP (*P* < 0.05) and increasing LVEF (*P* < 0.05), but the decrease of LVEDD in the rhBNP subgroup was similar to that of the dobutamine subgroup (*P* > 0.05) (Table 4).

**Table 4:** Changes in blood BNP, LVEDD, and LVEF in the high BNP group

**Parameters**	**Dobutamine (n = 30)**			**rhBNP (n = 28)**		
	**Baseline**	**5 Days**	**Changes**	**Baseline**	**5 Days**	**Changes**
BNP (pg/mL)	1890 ± 742	1261 ± 560^**^	629 ± 715	1819 ± 665	843 ± 450^**ΔΔ^	976 ± 566^Δ^
LVEDD (mm)	63.8 ± 4.6	61.2 ± 6.5^**^	2.6 ± 4.3	64.3 ± 4.9	60.9 ± 5.7^**^	3.3 ± 3.2
LVEF (%)	32.1 ± 6.1	39.6 ± 10.9^**^	7.5 ± 8.1	31.3 ± 6.8	43.7 ± 11.2^**^	12.4 ± 7.5^Δ^

*Abbreviations*: *LVEDD* left ventricle end diastolic dimension, *LVEF* left ventricular ejection fraction, Other abbreviations are same as in Table 2. Values are mean ± SD. ^**^P < 0.01 (paired *t*- test) versus baseline; ^Δ^P < 0.05, ^ΔΔ^P < 0.01 (unpaired *t*- test) versus dobutamine.

### Changes in blood BNP, LVEDD and LVEF in the extra-high BNP group

The measurement range for the ARCHITECT BNP assay is 10–5000 pg/mL. In the extra-high BNP group, there were 10 patients (6 from the dobutamine subgroup and 4 from the rhBNP subgroup) whose plasma BNP levels were >5000 pg/mL before the study; therefore, as we only selected patients with plasma BNP levels ≤5000 pg/mL for statistical analyses of BNP changes, their BNP levels could not be used for calculating the mean BNP values. Nonetheless, the changes in LVEDD and LVEF were still analyzed in all enrolled patients. Of the 10 patients with plasma BNP >5000 pg/mL, there were 5 and 3 patients in the dobutamine and rhBNP subgroups, respectively, whose plasma BNP levels were reduced to <5000 pg/mL. The average plasma BNP level in the rhBNP subgroup was slightly, but not significantly, higher than that of the dobutamine subgroup (4020 ± 710 pg/mL vs. 3508 ± 654 pg/mL; *P* > 0.05) at day 5 after treatment. Similarly, the BNP levels in patients with plasma BNP ≤ 5000 pg/mL was effectively reduced by rhBNP and dobutamine (*P* < 0.01) (Table 5). In addition, both rhBNP and dobutamine slightly, but significantly decreased the LVEDD (*P* < 0.05), and significantly increased the LVEF (*P* < 0.05) of all enrolled patients. The therapeutic efficacy of dobutamine in improving these parameters tended to be better than that of rhBNP, although the difference was not statistically significant (*P* > 0.05) (Table 5).

**Table 5:** Changes in blood BNP, LVEDD, and LVEF in the extra-high BNP group

**Parameters**	**Dobutamine (n = 23)**			**rhBNP (n = 23)**		
	**Baseline**	**5 Days**	**Changes**	**Baseline**	**5 Days**	**Changes**
BNP (pg/mL)	3790 ± 740	2806 ± 859^**^	985 ± 931^§^	3912 ± 726	3207 ± 900^**^	705 ± 884^※^
LVEDD (mm)	66.6 ± 5.1	64.2 ± 5.1^*^	2.4 ± 4.6	67.7 ± 5.4	65.6 ± 6.3^*^	2.1 ± 4.2
LVEF (%)	24.4 ± 5.7	31.6 ± 6.9^**^	7.2 ± 6.1	23.8 ± 5.6	29.0 ± 6.5^**^	5.2 ± 7.1

Abbreviations are same as in Table 2 and Table 4. Values are mean ± SD. ^§^n = 17, ^※^n = 19. ^*^P < 0.05, ^**^P < 0.01 (paired *t*- test) versus baseline.

### Length of hospitalization and All cause mortality

No patients died during administration of dobutamine or rhBNP. One patient with dilated cardiomyopathy in the extra-high BNP group whose symptoms were much improved by dobutamine suffered sudden death due to ventricular fibrillation at 8 days after withdrawal of dobutamine infusion during hospitalization; we excluded this patient from analysis of hospitalization length. In the high BNP group, length of hospitalization for rhBNP-treated patients was significantly shorter than for patients treated with dobutamine (8.0 ± 1.9 versus 9.3 ± 2.2 days, P < 0.05). However, in the extra-high BNP group, the length of hospitalization was not significantly different between the two subgroups (13.0 ± 3.1 versus 12.2 ± 2.7 days, P > 0.05). All-cause mortality during hospitalization was similar between the dobutamine and rhBNP subgroups in the high and extra-high BNP groups (both P = 1.000).

### Effects of the study drugs on blood pressure, HR and plasma creatinine

Ambulatory changes in systolic blood pressure (SBP), diastolic blood pressure (DBP), and HR were monitored during infusion. The SBP, DBP, and HR values at the end of infusion and the levels of plasma creatinine at day 5 after treatment were measured. Both rhBNP and dobutamine did not significantly affect HR and plasma creatinine (*P* > 0.05). The systolic and diastolic pressure were significantly decreased by rhBNP (*P* < 0.01), while no significant changes were observed in dobutamine-treated patients (*P* > 0.05) (Table 6).

**Table 6:** Effects of the study drugs on blood pressure (mmHg), HR (bpm) and plasma creatinine (μmol/L)

**Parameters**	**High BNP group**				**Extra-high BNP group**			
	**Dobutamine (n = 30)**		**rhBNP (n = 28)**		**Dobutamine (n = 23)**		**rhBNP (n = 23)**	
	**Baseline**	**5 Days**	**Baseline**	**5 Days**	**Baseline**	**5 Days**	**Baseline**	**5 Days**
SBP	119.3 ± 12.7	121.3 ± 12.0	122.9 ± 11.9	115.4 ± 12.4**	114.8 ± 11.9	116.0 ± 12.5	118.6 ± 13.0	113.2 ± 13.2**
DBP	72.4 ± 6.8	74.3 ± 5.3	73.5 ± 7.7	69.6 ± 7.9**	70.7 ± 6.7	71.8 ± 6.6	71.9 ± 6.5	68.7 ± 6.6**
HR	77.6 ± 12.5	80.3 ± 13.1	81.8 ± 11.1	83.9 ± 11.5	83.2 ± 13.9	85.7 ± 13.0	82.3 ± 12.4	84.6 ± 9.3
Cr	84.4 ± 29.8	79.3 ± 20.4	78.5 ± 31.9	80.9 ± 26.3	95.4 ± 24.4	90.3 ± 23.3	98.6 ± 30.1	105.5 ± 32.9

*Abbreviations*: *HR* heart rate. Other abbreviations are same as in Table 1 and Table 2. Values are mean ± SD. ^**^P < 0.01 (paired *t*- test) versus baseline.

### Cardiovascular adverse events

The incidence of selected cardiovascular adverse events during administration was similar between the rhBNP- and dobutamine-treated patients. However, symptomatic hypotension tended to be more common in the rhBNP-treated patients than the dobutamine-treated patients, although the difference was not statistically significant (Table 7). In the extra-high BNP group, one patient who was initially enrolled to the rhBNP subgroup had to withdraw from this study due to symptomatic hypotension. We still included this patient in the statistical analysis of adverse events.

**Table 7:** Selected cardiovascular adverse events during drug administration

**Adverse events**	**High BNP group**			**Extra-high BNP group**		
	**Dobutamine (n = 30)**	**rhBNP (n = 28)**	**P value**	**Dobutamine (n = 23)**	**rhBNP (n = 24)**	**P value**
Symptomatic hypotension	0	0	1.000	0	1	1.000
Asymptomatic hypotension	0	2	0.229	0	3	0.234
Sustained VT	0	0	1.000	0	0	1.000
Nonsustained VT	2	0	0.492	2	1	0.609
VF	0	0	1.000	0	0	1.000
Sudden death	0	0	1.000	0	0	1.000
Cardiac arrest	0	0	1.000	0	0	1.000

*Abbrevations*: *VF* ventricular fibrillation, *VT* ventricular tachycardia, Other abbreviations are same as in Table 2. Fisher exact text was used.

## Discussion

Our study is the first to compare the effects of rhBNP and dobutamine on ADHF patients with different plasma BNP levels. The results revealed that rhBNP was not superior to the traditional inotropic agent dobutamine at improving cardiac function in patients with extra-high BNP levels (>3000 pg/mL). On the contrary, rhBNP-treated patients tended to be more likely to experience hypotension than dobutamine-treated patients.

Dobutamine, a dopamine derivative, produces positive inotropic and chronotropic effects on myocardium mainly through stimulation of β1-receptors and by causing a reflex decrease in sympathetic tone [1,10,11]. At low doses, dobutamine induces mild arterial vasodilatation, which augments stroke volume by reductions in afterload [4], and has little influence on HR, blood pressure, and cardiovascular adverse events.

BNP, a natriuretic peptide, is produced and released from the ventricles in response to increased wall stretch and tension [2]. It binds to the A-type natriuretic peptide receptor that is present on the surface of vascular smooth muscle and endothelial cells [2], resulting in activation of guanylate cyclase and the subsequent accumulation of intracellular cyclic GMP in target tissues [12]. Cyclic GMP, as a second messenger, mediates natriuretic, diuretic and smooth muscle relaxant effects, which lead to the therapeutic benefits of venous and arterial vasodilation and decreases in preload and afterload, resulting in increased cardiac output with improved dyspnea and systemic symptoms [12].

In addition, BNP also serves as a physiological antagonist of the renin-angiotensin-aldosterone system and the sympathetic nervous system [13]. It inhibits renin-aldosterone secretion, suppresses the sympathetic nervous system, and antagonizes the action of antidiuretic hormone in inducing antidiuresis, antinatriuresis, and vasoconstriction, leading to a decrease in systemic vascular resistance and cardiac pre- and afterload [4,14,15]. Thus, as a compensatory mechanism for heart failure, circulating BNP levels strongly correlate with the severity of heart failure [16,17].

rhBNP has the same 32 amino acid sequence and biological activity as endogenous BNP, with a mean terminal elimination half-life of 18–20 min [18,19], which means that it is almost completely cleared at 3 h post infusion withdrawal (approximately 9–10 elimination half-lives). Several studies indicated that BNP changes during the first 5 days of hospitalization had a very high prognostic value in patients with heart failure [20,21]. Accordingly, we assayed the plasma BNP levels at day 5 after treatment to evaluate the efficacy of rhBNP and dobutamine. At this time, the exogenous rhBNP had been completely eliminated and did not interfere with the measurement of the endogenous BNP level, allowing accurate assessment of the therapeutic efficacy of the study drugs.

Numerous studies reported that BNP levels reflect heart failure severity [16,17,22]. However, Law et al. found that extremely high BNP level ( > 3000pg/mL) did not correlate with heart failure severity when patients had complications such as neurological disorder, sepsis, or subarachnoid hemorrhage. The possible reasons might be that in these patients, an increase in sympathetic stimulation causes the hypothalamus to secrete excess vasopressin and BNP [9,23]. In our study, all patients with these confounders were excluded. Thus, BNP level might well reflect the status of heart function.

We found that rhBNP was more efficient than dobutamine at improving heart function in patients with BNP levels ≤3000 pg/mL. However, their efficacies were similar in patients with extra-high BNP levels. The difference might be explained by the following reasons. First, BNP exerts its biological activities through binding to the A-type natriuretic peptide receptor, which is present on the surface of vascular smooth muscle and endothelial cells [2]. When the compensatorily increased endogenous BNP is not sufficient to bind to all the free receptors, the introduced exogenous BNP (rhBNP) can fully activate the remaining free receptors, resulting in a marked improvement in heart function. However, when the circulating BNP increases to even greater levels, with subsequent full activation of their receptors, an insufficient number of free BNP receptors can be bound by exogenous BNP, meaning that the additional effects of exogenous BNP on decreasing cardiac pre- and afterload are inhibited. Second, patients with extremely high BNP levels might be resistant to the biological action of BNP. This phenomenon is characterized by extremely high circulating BNP levels in patients with congestive heart failure who have physical signs of fluid retention and vasoconstriction from poor biological activity of the BNP system [14]. A deficient response to BNP is attributed to inactivation of BNP by plasma and tissue proteases before they bind to their receptors, downregulation or desensitization of BNP-specific receptors, and mechanisms that counteract the biological effects of BNP at postreceptor level [14]. Third, patients with extremely high BNP levels usually suffer from more severe heart failure, accompanied by serious renal congestion and low renal blood flow [24], which could inhibit the effects of rhBNP on renal vessel dilation, natriuresis, and diuresis, but would not affect the positive inotropic effects of dobutamine on myocardium. In this study, basal plasma creatinine was significantly higher in the extra-high BNP group, suggesting deterioration of renal function might be responsible for the low efficiency of rhBNP in patients with extremely high BNP. In addition, patients with more severe heart failure usually also have lower arterial pressure. The SBP level in the extra-high BNP group tended to be lower than in the high BNP group, although the difference was not significant (P = 0.079). Vasodilator drugs such as rhBNP might lead to more satisfactory clinical improvements in heart failure patients with high-normal blood pressure.

The blood pressure in the rhBNP subgroup was decreased after treatment (Table 6). One patient in the extra-high BNP group with low baseline blood pressure (96/64 mmHg) experienced symptomatic hypotension (84/50 mmHg) at 3 h after rhBNP infusion and had to withdraw from the study. Plasma creatinine remained unchanged in patients with plasma BNP ≤3000 pg/mL after treatment, but it tended to increase in patients with extra-high BNP levels after rhBNP treatment (Table 6). One male patient in the extra-high BNP group who received rhBNP had an increase in plasma creatinine from 165 μmol/L (1.9 mg/dl, baseline) to 243 μmol/L (2.7 mg/dl). These results indicate that rhBNP, as an antagonist of the renin-angiotensin-aldosterone system (RAAS), might impair renal function and induce azotemia in patients with severe heart failure whose renal function depends on RAAS. Dobutamine had no obvious effect on plasma creatinine.

### Study limitations

The results of this study are limited by its open-label design and the relatively small number of patients in each subgroup. Moreover, the effects of rhBNP and dobutamine on hospital readmissions and long-term survival were not assessed. More information from a larger blinded study will be required to confirm the results of this study.

## Conclusions

We found that rhBNP was more efficient than dobutamine in ADHF patients who had plasma BNP ≤3000 pg/mL. However, rhBNP therapy was not superior to dobutamine when BNP reached extremely high level (>3000 pg/mL).

## Abbreviations

ADHF: Acute decompensated heart failure; CHD: Coronary heart disease; DBP: Diastolic blood pressure; FVM: Fulminant viral myocarditis; HHD: Hyperthyroid heart disease; HR: Heart rate; IDC: Idiopathic dilated cardiomyopathy; LVEDD: Left ventricular end diastolic diameter; LVEF: Left ventricular ejection fraction; NYHA: New York Heart Association; RAAS: Renin-angiotensin-aldosterone system; rhBNP: Recombinant human B-type natriuretic peptide; SBP: Systolic blood pressure.

## Competing interests

The authors declare that they have no competing interests.

## Authors’ contributions

PHY conceived and designed the study, analyzed and interpreted the data, drafted the manuscript and critically revised the manuscript for intellectual content. ZJH was responsible for acquisition, analysis and interpretation of data, drafting of the manuscript, and final approval of the published version. GY was responsible for analysis and interpretation of data and critical revision of the manuscript. YXH and PM inspected and approved the final version of the manuscript. NHY was responsible for analysis and interpretation of data. All authors read and approved the final manuscript.

## Pre-publication history

The pre-publication history for this paper can be accessed here:

http://www.biomedcentral.com/1471-2261/14/31/prepub
